# Positive Attitude toward Healthy Eating Predicts Higher Diet Quality at All Cost Levels of Supermarkets^☆^

**DOI:** 10.1016/j.jand.2013.06.006

**Published:** 2014-02

**Authors:** Anju Aggarwal, Pablo Monsivais, Andrea J. Cook, Adam Drewnowski

**Keywords:** Attitude toward healthy eating, Supermarket access and food environment, Cost level of supermarkets, Diet quality, Fruit and vegetable intake

## Abstract

Shopping at low-cost supermarkets has been associated with higher obesity rates. This study examined whether attitudes toward healthy eating are independently associated with diet quality among shoppers at low-cost, medium-cost, and high-cost supermarkets. Data on socioeconomic status (SES), attitudes toward healthy eating, and supermarket choice were collected using a telephone survey of a representative sample of adult residents of King County, WA. Dietary intake data were based on a food frequency questionnaire. Thirteen supermarket chains were stratified into three categories: low, medium, and high cost, based on a market basket of 100 commonly eaten foods. Diet-quality measures were energy density, mean adequacy ratio, and total servings of fruits and vegetables. The analytical sample consisted of 963 adults. Multivariable regressions with robust standard error examined relations between diet quality, supermarket type, attitudes, and SES. Shopping at higher-cost supermarkets was associated with higher-quality diets. These associations persisted after adjusting for SES, but were eliminated after taking attitudinal measures into account. Supermarket shoppers with positive attitudes toward healthy eating had equally higher-quality diets, even if they shopped at low-, medium-, or high-cost supermarkets, independent of SES and other covariates. These findings imply that shopping at low-cost supermarkets does not prevent consumers from having high-quality diets, as long as they attach importance to good nutrition. Promoting nutrition-education strategies among supermarkets, particularly those catering to low-income groups, can help to improve diet quality.

The selection of high-quality diets is influenced by knowledge, attitudes, and the economics of food-choice behavior.^1-6^ Positive food-related attitudes have been linked to better diets, as indexed by higher values of the Healthy Eating Index and by higher consumption of vegetables and fruit.^7^ Diet quality has also been linked to the food environment.^8-13^ Improved access to full-service supermarkets has been associated with better diets and with higher consumption of vegetables and fruit.^14,15^ Ensuring equal access to supermarkets has thus recently become a focus of public health policy.^16,17^

However, a recent study found a threefold variation in obesity even among adults who primarily shopped at supermarkets. Lower-cost supermarkets, in particular, were linked to higher obesity rates.^18^ Social class or unmeasured attitudinal factors were proposed to explain this phenomenon. The purpose of the present study was to examine whether there exists a gradient in diet quality among supermarket shoppers, and the extent to which the variability in food-related attitudes and socioeconomic status (SES) can explain this relation. We hypothesized that shopping at lower-cost supermarkets would be associated with lower diet quality and this relationship would be explained by SES and attitude toward healthy foods. The question was whether shoppers at lower-cost supermarkets achieve high-quality diets if they attach importance to good nutrition. The present study was unique in collecting individual-level data on actual food shopping destinations by supermarket chain brand, availability and prices of commonly consumed foods at each of these supermarkets, food-related attitudes, and sociodemographic characteristics, which allowed tests of these hypotheses.

## Methods

### Participant Sample

Data were collected as part of the Seattle Obesity Study, using a stratified random sample of 2,001 adult residents of King County, WA, conducted in 2008-2009. Details on sampling and study procedures have been published previously.^18-20^ The Seattle Obesity Study combined telephone survey procedures, based on the Behavioral Risk Factor Surveillance System survey, with a mailed food frequency questionnaire (FFQ). A 20-minute telephone survey was used to collect data on food-shopping behaviors, names and locations of food stores frequented by respondents, food-related attitudes, and sociodemographic characteristics. Telephone survey respondents were asked to complete an additional dietary intake assessment. The majority (n=1,903 [95%]) agreed and FFQs were mailed to their home addresses. Completed FFQs (n=1,318) were checked for missing data and were sent to the Fred Hutchinson Cancer Research Center for processing. After removing respondents with outliers in calorie intakes (14 respondents with calorie intake <500 kcal/day and 9 respondents with >5,000 kcal/day) and respondents with missing data for key variables of interest (ie, income, education, and supermarket type), the final analytical sample consisted of 963 respondents. The sociodemographic profile of the total sample (2,001 respondents) was compared with the sample available for analyses (963 respondents). The analytical sample tended to have more white respondents (86% vs 80%) and more college-educated respondents (58% vs 54%). However, no significant differences were seen in other sociodemographic variables. The University of Washington Institutional Review Board approved study protocols and informed consent was obtained from study respondents.

### Variable Definitions

#### Socioeconomic and Demographic Measures

Demographic variables of interest were age, sex, and race/ethnicity. Six categories of education were recoded: “high school,” “some college,” and “≥college degree.” Annual household income was categorized into: “<$50K,” “≥$50 to <100K,” and “≥$100K.” Household size was a covariate during analyses. A five-category index of SES was also created to capture the combined effects of income and education.^21,22^ The categories ranged from lower income and lower education (income <$50K and <college degree) to highest income and higher education (income ≥$100K and ≥college degree) (see Table 1 footnote).

### Dietary-Intake Data and Diet-Quality Measures

Dietary data were collected using the General Select version of the Fred Hutchinson Cancer Research Center FFQ,^23^ a modified version of the FFQ used previously in Women's Health Initiative studies.^24-26^ Participants indicated, for the past year, the frequency of consumption of each food and beverage with portion size. Detailed methodology on analyses of FFQ dietary data have been published previously.^27^ Analyses yielded dietary energy (kcal), weight of foods and beverages consumed, estimated daily intakes of >45 macro- and micronutrients, as well as food-based indices, such as fruit and vegetable intake, all at the individual level.

Diet-quality measures were energy density, mean adequacy ratio (MAR), and fruit and vegetable intakes.^6,7,19,27,28^ Energy density is the ratio of total calorie intake to daily weight of foods and caloric beverages consumed (kcal/g). MAR is the average of the truncated Nutrient Adequacy Ratios for 11 key nutrients in the diet (ie, vitamin A, C, D, E, B-12, calcium, iron, magnesium, potassium, folate, and fiber). The truncated Nutrient Adequacy Ratio for each nutrient was defined as the daily nutrient intake divided by the age- and sex-specific dietary reference intake,^29^ with a maximum value of 1 (so that intake of any nutrient that exceeds the daily reference intake cannot mask lower intakes of other nutrients).^30^ Total servings of fruits and vegetables per day were the food-based index of diet quality.^31^

### Identification of the Primary Supermarket Reported

During the telephone survey, Seattle Obesity Study respondents were asked “what is the name of the primary store where your household purchases most of the foods you eat?” Participants also reported the exact store location, frequency of shopping trips, the amount spent at this store per visit, and monthly household food expenditures. These questions were repeated for one secondary food store as well.

Self-reported data on primary food stores was used solely to identify the primary supermarket for each respondent. These stores accounted for approximately 70% of the household monthly food expenditure and were visited more often (at least two to three times per week). Of the primary stores reported in the present sample, 92% were supermarkets, which constituted the sample for the present analyses. Of these, 88% were the eight supermarkets with maximum penetration in the King County area.

### Classification of Primary Supermarkets by Price

To collect data on availability and prices at 13 primary supermarkets reported by respondents, a market basket of 100 commonly consumed foods was developed. The detailed procedures for market-basket data collection have been published previously.^18,32,33^ In brief, market-basket data were collected for eight primary supermarkets through in-person visits. Five additional supermarkets were reported by the remaining 12% of the sample, prices for which were collected through store websites and contact with store managers. Results showed that the availability of foods was close to 100% at each of these supermarkets; however, there was a considerable variation by price.^33^ We used cluster analysis to stratify supermarkets by price. The low-cost strata consisted of five supermarkets with an average market-basket cost of $224, the medium-cost strata consisted of four supermarkets with an average cost of $305 (30% to 40% more expensive), and the high-cost strata consisted of remaining four supermarkets with an average market basket cost of $393 (70% more expensive). Prices were collected from January 2009 through April 2009, which coincided with the period of data collection from other study instruments.

### Attitude toward Healthy Eating

Participants in the telephone survey were read the following statement: “It is important to me that the foods I usually eat are healthy _____” and were asked to respond on a 5-point Likert scale ranging from “strongly disagree” to “strongly agree.” This question is analogous to the one used in the National Health and Nutrition Examination Survey's Flexible Consumer Behavior Module^34^ and in many health studies. For analytical purposes, the variable was recoded based on the distribution of data obtained. Although most of the respondents either strongly agreed (61%) or somewhat agreed (34%) with the importance of eating healthy foods, a relatively small proportion chose the neutral (3%), somewhat disagreed (2%), or strongly disagreed category (0.4%). Due to the limited number of responses, the neutral and disagreeing groups were combined together. (Sensitivity analyses were also conducted and no statistically significant differences were observed between neutral and disagreeing groups by the diet-quality outcomes variables.) The three analytical attitude categories were highly positive (those who strongly agreed), somewhat positive (somewhat agreed group), and neutral/negative (neutral/somewhat disagreed/strongly disagreed group).

### Statistical Methods

Sample characteristics were described using means and proportions for continuous and categorical variables, respectively. Diet-quality measures were energy density, MAR, and daily servings of fruits and vegetables. Bivariate regression analyses with robust standard errors^35^ were used to examine the associations of each: supermarket type, SES, and attitudes toward healthy eating with diet quality.

Multivariable regressions with robust standard errors examined associations between supermarket type and diet quality, before and after adjusting for SES and the attitude variable. Each diet-quality measure was used as the dependent variable, and supermarket type was the main independent variable. Other covariates included age, sex, race/ethnicity, household size, and total calorie intake. Adjusted mean diet quality was expressed using mean age (56 years) and mean calorie intake (1,800 kcal/day) for the sample.

Multivariable regressions with robust standard errors were repeated to examine associations between attitude variable and diet quality before and after stratifying by supermarket type. The sample was stratified based on the cost level of the supermarket participants primarily used (ie, lower, medium, and higher cost). Diet-quality measures were dependent variables and the attitude variable was the main independent variable. Other covariates included age, sex, race/ethnicity, household size, SES index, and total calorie intake. Likelihood ratio tests were also conducted to test the interaction between supermarket type and attitudes. However, due to limited power, the results focused on the effect estimates presented in the stratified tables. An α level of .05 was used to determine statistical significance. All analyses were conducted using STATA Statistical Software, release 10 (StataCorp LP).

## Results and Discussion

The sample was 63% women and 37% men. Mean age was 56 years. The sample was mostly white (85%) with 5% African Americans and 7% Asians. Annual household income was ≥$50K for 60% of the sample. Most participants (58%) were college graduates. Other participant characteristics are summarized in Table 1.

Bivariate analyses showed that SES variables, education and income, shopping at higher-cost supermarkets, and positive attitudes toward healthy eating were each associated with better diets, as indexed by lower dietary energy density, higher MAR, and more servings of fruits and vegetables (Table 1).

### Differences in Diet Quality by Supermarket Type

Multivariate analyses, adjusted for demographics, showed that shopping at higher-cost supermarkets was associated with lower dietary energy density (mean energy density was 0.04 kcal/g lower among high-cost supermarket shoppers as compared with low cost), higher MAR scores (approximately 4 units higher among high-cost supermarket shoppers), and more daily servings of fruits and vegetables (0.93 servings higher) (Table 2, Model 1). These associations were highly significant for MAR and fruit and vegetable intakes (*P*<0.05) and near significant for energy density (*P*=0.069). Taking SES into account attenuated the observed associations between supermarket type and diet-quality variables (Model 2). The mean energy density remained 0.03 kcal/g lower among high-cost supermarket shoppers vs low-cost supermarkets, mean MAR remained only one unit higher among high-cost supermarket shoppers, and mean daily intake of fruits and vegetables was 0.70 servings higher (Model 2). However, taking variability in the attitude variable into account (Model 3) completely eliminated the supermarket effect for energy density and MAR (dietary energy density and MAR were the same among high-cost and low-cost supermarket shoppers) and mean daily intake of fruits and vegetables was only 0.3 servings higher among high-cost than among low-cost supermarket shoppers (*P*>0.05) (Model 3).

### Differences in Diet Quality by Attitude Variable

As shown in Table 3, positive attitudes toward healthy eating were associated with higher diet quality, adjusting for sociodemographic variables. A dose−response relation was observed across all the diet-quality measures. For example, compared to those with a neutral/negative attitude, mean daily intake of fruits and vegetables was approximately 1.13 servings higher among those with a somewhat positive attitude and 2.25 servings higher among those with a highly positive attitude toward healthy eating. Further, positive attitudes toward healthy eating were not restricted to shoppers at high-cost supermarkets. Shoppers with positive attitudes also shopped at low- and medium-cost supermarkets. Additional analyses were conducted to examine whether positive attitudes toward healthy eating were associated with equally higher diet quality among low-, medium-, and high-cost supermarket shoppers.

### Differences in Diet Quality by Attitude at Each Supermarket Cost Level

For each supermarket type, positive attitudes toward healthy eating were consistently and significantly associated with lower dietary energy density, higher MAR scores, and more daily servings of fruits and vegetables, adjusting for sociodemographic variables and calorie intake (Table 3). For example, within low-cost supermarket patrons, those shoppers with highly positive attitudes had fruit and vegetable intakes that were twice as high as shoppers who had neutral/negative attitudes (5.27 vs 2.83 servings/day). Similar trends were seen with other diet-quality measures. In addition, the magnitude of the difference in diet quality with differences in attitudes was similar across all cost levels of supermarket type (*P* value for interactions >0.303 for all outcomes).

To summarize, the present study had three major findings. First, a significant gradient in diet quality was observed even among those who used supermarkets for their primary food shopping. Those shopping at higher-cost supermarkets had higher-quality diets as compared with those shopping at lower-cost supermarkets. Second, although some of these observed differences in diet quality were reduced after accounting for variability in SES, the differences were completely eliminated after taking attitude into account. These findings imply that improving physical access to supermarkets is insufficient to improve diet quality unless underlying socioeconomic and psychosocial determinants (eg, prioritizing the importance of healthy eating, and providing the socioeconomic support for such prioritization) are addressed. Third, those with positive attitudes toward healthy eating achieved higher-quality diets within each cost level of supermarkets, independent of SES. Even among shoppers of lower-cost supermarkets, those who prioritized healthy eating achieved higher-quality diets. This indicates that shopping at low-cost supermarkets is not a barrier to achieving higher-quality diets when consumers attach importance to good nutrition. Ensuring economic access to healthy foods and motivating consumers to seek out healthier food choices through continued nutrition education are equally important strategies for improving diets in the population.

The present study had several strengths. Although numerous past studies have linked food-related attitudes to diet quality, they lacked data on the economics of food-shopping behavior.^7,36-38^ By contrast, studies on diets and the built environment did not have access to data on food-related attitudes.^10,11^ The present study was the combination of psychosocial, geographic, and economic data on food-acquisition patterns, all collected at the individual level. This allowed the linking of data on shopping behaviors, food-related attitudes, and diet quality.

However, it has certain limitations as well. First, diet-quality variables were obtained from FFQ, which might not represent the actual intakes for respondents due to certain known biases.^39,40^ However, FFQ has been useful in making comparisons across subjects and, therefore, the results from the present study were not biased. Second, physical access to supermarkets and availability of healthy foods across supermarkets did not appear to be a problem in the present sample. The present findings might not be generalizable to other areas of the United States with different availability of supermarkets and/or healthy foods. Third, classification of respondents into a supermarket type was based solely on one self-reported primary supermarket. As these stores accounted for most of the household food purchases (approximately 70%), we propose that the present sample was not strongly misclassified by using this criterion. However, more detailed data on food-acquisition patterns might be needed among populations using more than one store for food shopping on a regular basis. Fourth, the food-related attitude variable was based on one question and might have been affected by social desirability bias. In addition, this bias might have differed at different levels of SES. However, this is a standard question used in national-level surveys and other health studies. Fifth, the present findings are based on cross-sectional data, which limits our ability to draw causal inferences. Finally, the distribution of respondents by attitude was not optimal in the present sample: only 5% had neutral/negative attitudes toward healthy eating. However, our conclusions remained the same, even among those groups with somewhat positive vs highly positive attitudes. We propose that the present findings would get even stronger with broader representation of the attitude variable.

## Conclusions

The finding that attitude toward healthy eating was among the key predictors of diet quality among supermarket shoppers, and that it persisted at every cost level of the supermarket, independent of SES, has many practical applications. Ensuring physical access to supermarkets has recently become one strategy to improve diet quality among vulnerable groups.^41,42^ At the same time, consumers need to be motivated to make healthy food choices once inside the supermarket. Public health policies on improving physical access to healthy and affordable foods need to be coupled with continuing nutrition education about the importance of healthy eating. In particular, supermarkets that cater to lower-income groups might need to develop nutrition-education strategies to create increased demand for affordable and appealing nutrient-dense foods.

## Figures and Tables

**Table 1:** Sample characteristics and crude mean±standard error of diet quality measures^a^ by socioeconomic status (SES) indicators, supermarket type, and attitude toward of healthy eating

Characteristics	Total, n (%)	Energy density (kcal/g)	MAR^b^(% adequacy/day)	Total servings of fruits+vegetables/day
		← *mean±standard error*→		
**Overall**		1.15±0.27	76±16	4.53±2.51
**Annual household income ($)**				
<50,000	374 (39)	1.14±0.01	74±0.72	4.45±0.16
≥50,000 to <100,000	334 (35)	1.12±0.02	76±0.83	4.84±0.20
≥100,000	255 (26)	1.10±0.02	77±1.01	4.66±0.22
**Education**				
High school or less	162 (17)	1.16±0.02	71±0.99	4.11±0.22
Some college	241 (25)	1.14±0.02	74±0.86	4.38±0.20
College graduate or higher	560 (58)	1.11±0.02	77±0.73	4.92±0.17
**SES**^c^**Index**				
Category 1	224 (23)	1.14±0.02	72±0.85	4.08±0.19
Category 2	179 (19)	1.15±0.02	74±1.05	4.45±0.23
Category 3	150 (15)	1.14±0.02	76±0.95	4.97±0.22
Category 4	211 (22)	1.08±0.02	78±0.86	5.11±0.22
Category 5	199 (21)	1.10±0.02	78±1.06	4.59±0.23
**Supermarket type by price**				
Low cost	306 (31)	1.16±0.01	75±0.58	4.35±0.12
Medium cost	545 (57)	1.14±0.01	76±0.47	4.43±0.10
High cost	112 (12)	1.12±0.02	79±0.82	5.25±0.22
**Attitude toward healthy eating**				
Neutral/negative	49 (5)	1.33±0.05	67±1.69	2.33±0.19
Somewhat positive	329 (34)	1.18±0.01	74±0.57	3.77±0.10
Highly positive	585 (61)	1.11±0.01	78±0.40	5.09±0.09

a Each adjusted for total calorie intake. Means expressed at mean calorie intake of 1,800 kcal/day for the sample.

b MAR=mean adequacy ratio.

c Socioeconomic index defined in 5 categories: Category 1: low income and low education (income <$50K and <college graduates), Category 2: higher income and low education (income ≥$50K and <college graduates), Category 3: low income and high education (income <$50K and ≥college graduates), Category 4: higher income and high education (income ≥$50K to <100K and ≥college graduates), Category 5: highest income and high education (income ≥$100K and ≥college graduates).

**Table 2:** Adjusted mean±standard error diet quality by supermarket type used, before and after taking socioeconomic status and attitude toward healthy eating into account

Characteristics	Energy density (kcal/g)	MAR^a^ (% adequacy/day)	Total servings of fruits+vegetables consumed/day
**Supermarket type frequented (Model 1**^b^**)**	←*mean±standard error*→		
Lower cost	1.15±0.01	78±0.60	4.81±0.14
Medium cost	1.13±0.01	79±0.51	4.95±0.11
Higher cost	1.11±0.02	82±0.79	5.74±0.22
	←P *value for trend test*→		
	0.069	0.011	0.002
**Supermarket type frequented (Model 2**^c^**)**	←*mean±standard error*→		
Lower cost	1.15±0.02	75±1.05	4.19±0.26
Medium cost	1.14±0.02	75±0.96	4.28±0.24
Higher cost	1.12±0.03	76±1.23	4.90±0.32
	←P *value for trend test*→		
	0.254	0.252	0.023
**Supermarket type frequented (Model 3**^d^**)**	←*mean±standard error*→		
Lower cost	1.11±0.02	76±1.08	4.74±0.26
Medium cost	1.11±0.02	76±0.97	4.78±0.24
Higher cost	1.11±0.03	76±1.21	5.04±0.31
	←P *value for trend test*→		
	0.709	0.907	0.308

a MAR=mean adequacy ratio.

b Model 1: Adjusted for age+sex+race/ethnicity+household size+total calorie intake. Means expressed at mean age of 56 years and mean calorie intake of 1,800 kcal/day for the sample.

c Model 2: Adjusted for Model 1+socioeconomic status index. Means expressed at mean age of 56 years and mean calorie intake of 1,800 kcal/day for the sample.

d Model 3: Adjusted for Model 2+attitudes toward healthy foods. Means expressed at mean age of 56 years and mean calorie intake of 1,800 kcal/d for the sample.

**Table 3:** Adjusted^a^ mean±standard error of diet-quality measures by attitude toward healthy eating, before and after stratifying by supermarket type

Independent variables	n	Energy density (kcal/g)	MAR^b^ (% adequacy/day)	Total servings of fruits+vegetables/day
		←*mean±standard error*^c^→		
**Attitude toward healthy eating**				
Neutral or negative	49	1.31±0.06∗	67±1.78∗∗∗	2.55±0.39∗∗
Somewhat positive	329	1.17±0.02∗	73±0.99∗∗∗	3.68±0.24∗∗
Strongly positive	585	1.11±0.02∗∗	76±0.95∗∗∗	4.80±0.23∗∗∗
**After stratifying by supermarket type**				
Among low-cost supermarket patrons				
Attitude toward healthy eating				
Neutral or negative	16	1.21±0.07	68±3.25∗	2.83±0.47∗∗∗
Somewhat positive	122	1.15±0.04	74±1.69∗	4.08±0.37∗∗∗
Strongly positive	168	1.08±0.04∗	77±1.67∗	5.27±0.44∗∗∗
Among medium-cost supermarket patrons				
Attitude toward healthy eating				
Neutral or negative	33	1.34±0.08∗	67±2.14∗	2.46±0.54∗
Somewhat positive	191	1.18±0.04∗	72±1.33∗	3.53±0.37∗
Strongly positive	321	1.11±0.03∗	76±1.25∗∗∗	4.55±0.32∗∗∗
Among high-cost supermarket patrons				
Attitude toward healthy eating				
Neutral or negative	0	–	–	–
Somewhat positive	16	1.21±0.13	79±3.86	3.33±0.89∗∗∗
Strongly positive	96	1.11±0.09	82±3.22	5.21±0.80∗∗∗

a Adjusted for age+sex+race/ethnicity+socioeconomic index+household size+calorie intake. Means expressed at mean age of 56 years and mean calorie intake of 1,800 kcal/day.

b MAR=mean adequacy ratio.

c Asterisk indicates strength of pairwise statistical significance. Out of three categories, those marked with an asterisk are statistically significant from the other two. The number of asterisks indicate the level of significance:

∗ *P*<0.05.

∗∗ *P*<0.005.

∗∗∗ *P*<0.0001. If a category is not marked with an asterisk, it indicates that it is not statistically different from the other two categories.

## References

[1] Darmon N., Drewnowski A. (2008). Does social class predict diet quality?. Am J Clin Nutr.

[2] Kant A.K., Graubard B.I. (2007). Secular trends in the association of socio-economic position with self-reported dietary attributes and biomarkers in the US population: National Health and Nutrition Examination Survey (NHANES) 1971-1975 to NHANES 1999-2002. Public Health Nutr.

[3] Moser R.P., Green V., Weber D., Doyle C. (2005). Psychosocial correlates of fruit and vegetable consumption among African American men. J Nutr Educ Behav.

[4] Gittelsohn J., Anliker J.A., Sharma S., Vastine A.E., Caballero B., Ethelbah B. (2006). Psychosocial determinants of food purchasing and preparation in American Indian households. J Nutr Educ Behav.

[5] Glanz K., Basil M., Maibach E., Goldberg J., Snyder D. (1998). Why Americans eat what they do: Taste, nutrition, cost, convenience, and weight control concerns as influences on food consumption. J Am Diet Assoc.

[6] Beydoun M.A., Wang Y. (2008). Do nutrition knowledge and beliefs modify the association of socio-economic factors and diet quality among US adults?. Prev Med.

[7] Beydoun M.A., Wang Y. (2008). How do socio-economic status, perceived economic barriers and nutritional benefits affect quality of dietary intake among US adults?. Eur J Clin Nutr.

[8] Laraia B.A., Siega-Riz A.M., Kaufman J.S., Jones S.J. (2004). Proximity of supermarkets is positively associated with diet quality index for pregnancy. Prev Med.

[9] Michimi A., Wimberly M.C. (2010). Associations of supermarket accessibility with obesity and fruit and vegetable consumption in the conterminous United States. Int J Health Geogr.

[10] Morland K., Wing S., Roux A.D. (2002). the contextual effect of the local food environment on residents' diets: The Atherosclerosis Risk in Communities Study. Am J Public Health.

[11] Rose D., Richards R. (2004). Food store access and household fruit and vegetable use among participants in the US Food Stamp Program. Public Health Nutr.

[12] Moore L.V., Diez Roux A.V., Nettleton J.A., Jacobs D.R. (2008). Associations of the local food environment with diet quality—A comparison of assessments based on surveys and geographic information systems: The multi-ethnic study of atherosclerosis. Am J Epidemiol.

[13] Popkin B.M., Duffey K., Gordon-Larsen P. (2005). Environmental influences on food choice, physical activity and energy balance. Physiol Behav.

[14] Treuhaft S., Karpyn A. (2010). The Grocery Gap: Who Has Access to Healthy Food and Why It Matters.

[15] Larson N., Story M., Nelson M. (July 2008). Bringing Healthy Foods Home: Examining Inequalities in Access to Food Stores.

[16] Ploeg M.V. (2010). Access to Affordable, Nutritious Food Is Limited in “Food Deserts”.

[17] Task Force on Childhood Obesity. *Solving the Problem of Childhood Obesity Within a Generation: Access to Healthy, Affordable Food*. http://www.letsmove.gov/white-house-task-force-childhood-obesityreport-president. Accessed June 5, 2012.

[18] Drewnowski A., Aggarwal A., Hurvitz P.M., Monsivais P., Moudon A.V. (2012). Obesity and supermarket access: Proximity or price?. Am J Public Health.

[19] Aggarwal A., Monsivais P., Cook A.J., Drewnowski A. (2011). Does diet cost mediate the relation between socioeconomic position and diet quality?. Eur J Clin Nutr.

[20] Moudon A.V., Cook A., Ulmer J., Hurvitz P., Drewnowski A. (2011). Wealth and health: A property values metric of SES for use in health studies. Am J Prev Med.

[21] Turrell G., Hewitt B., Patterson C., Oldenburg B. (2003). Measuring socio-economic position in dietary research: Is choice of socio-economic indicator important?. Public Health Nutr.

[22] Panagiotakos D.B., Pitsavos C., Chrysohoou C. (2008). Dietary habits mediate the relationship between socio-economic status and CVD factors among healthy adults: The ATTICA study. Public Health Nutr.

[23] Fred Hutchinson Cancer Research Center (FHCRC). *Food Frequency Questionnaire (GSEL)*. FHCRC Nutrition Assessment Shared Resource, Seattle, WA. http://sharedresources.fhcrc.org/services/food-frequency-questionnaires-ffq. Accessed February 20, 2012.

[24] Patterson R.E., Kristal A.R., Tinker L.F., Carter R.A., Bolton M.P., Agurs-Collins T. (1999). Measurement characteristics of the Women's Health Initiative food frequency questionnaire. Ann Epidemiol.

[25] Neuhouser M.L., Kristal A.R., McLerran D., Patterson R.E., Atkinson J. (1999). Validity of short food frequency questionnaires used in cancer chemoprevention trials: Results from the Prostate Cancer Prevention Trial. Cancer Epidemiol Biomarkers Prev.

[26] Jackson R.D., LaCroix A.Z., Gass M. (2006). Calcium plus vitamin D supplementation and the risk of fractures. N Engl J Med.

[27] Monsivais P., Drewnowski A. (2009). Lower-energy-density diets are associated with higher monetary costs per kilocalorie and are consumed by women of higher socioeconomic status. J Am Diet Assoc.

[28] Madden J.P., Goodman S.J., Guthrie H.A. (1976). Validity of the 24-hr. recall. Analysis of data obtained from elderly subjects. J Am Diet Assoc.

[29] Food and Nutrition Board, Institute of Medicine (2004). Dietary Reference Intakes: Recommended Intakes for Individuals.

[30] Maillot M., Darmon N., Vieux F., Drewnowski A. (2007). Low energy density and high nutritional quality are each associated with higher diet costs in French adults. Am J Clin Nutr.

[31] Fred Hutchinson Cancer Research Center (FHCRC). *FFQ Technical documentation (GSEL)*. FHCRC Nutrition Assessment Shared Resource. http://sharedresources.fhcrc.org/content/technical-documentation-gsel. Accessed February 20, 2012.

[32] Monsivais P., Drewnowski A. (2007). The rising cost of low-energy-density foods. J Am Diet Assoc.

[33] Mahmud N.K., Monsivais P., Drewnowski A. (July 2009). The Search for Affordable Nutrient Rich Foods: A Comparison of Supermarket Food Prices in Seattle-King County.

[34] Center for Disease Control and Prevention (August 2007). National Health and Nutrition Examination Survey (NHANES): Flexible Consumer Behavior Survey (FCBS) Module.

[35] White H.A. (1980). heteroskedasticity-consistent covariance matrix estimator and a direct test for heteroskedasticity. J Econometric Soc.

[36] Williams L., Ball K., Crawford D. (2010). Why do some socioeconomically disadvantaged women eat better than others? An investigation of the personal, social and environmental correlates of fruit and vegetable consumption. Appetite.

[37] Gittelsohn J., Sharma S. (2009). Physical, consumer, and social aspects of measuring the food environment among diverse low-income populations. Am J Prev Med.

[38] Havas S., Treiman K., Langenberg P. (1998). Factors associated with fruit and vegetable consumption among women participating in WIC. J Am Diet Assoc.

[39] Willett W. (1998). Nutritional Epidemiology. Vol 30.

[40] Kristal A.R., Peters U., Potter J.D. (2005). Is it time to abandon the food frequency questionnaire?. Cancer Epidemiol Biomarkers Prev.

[41] Let's Move Campaign. http://www.letsmove.gov/accessing/index.html. Accessed May 15, 2010.

[42] The Food Trust. *Stimulating Supermarket Development: A New Day for Philadelphia*. 2004. http://www.thefoodtrust.org/php/reports/archive.php. Accessed June 5, 2012.

